# Clinical Application of Noninflating Video-Endoscopic Inguinal Lymph Node Dissection

**DOI:** 10.1155/2022/8259990

**Published:** 2022-06-28

**Authors:** Jinhu Chen, Lei Yan, Guangyue Luo, Weihua Fang, Chaozhao Liang

**Affiliations:** ^1^Department of Urology, The First Affiliated Hospital of Anhui Medical University, China; ^2^Institute of Urology, Anhui Medical University, China; ^3^Anhui Province Key Laboratory of Genitourinary Diseases, Anhui Medical University, Hefei 230022, China

## Abstract

**Objective:**

To assess the safety and efficacy of the application of self-made non-inflating suspension technique in video endoscopic inguinal lymph node dissection (ILND).

**Methods:**

We collected 8 patients with penile carcinoma who underwent noninflating video-endoscopic ILND in the Department of Urology, the First Affiliated Hospital of Anhui Medical University, from May 2019 to March 2021. Then, surgical duration, blood loss, drainage tube indwelling time, hospital stay, number of dissected lymph nodes, and complications in the patients were analyzed.

**Results:**

All patients (*n* = 8) finished the surgery successfully, with an average surgical duration of 125 (105-145) minutes, blood loss of 41 (25-50) mL, indwelling time of drainage tube of 7 (5-12) days, and a hospital stay of 14.8 (9-21) days. Additionally, 8.8 (3-14) left side and 7.3 (2-17) right side lymph nodes were dissected on average. Complications occurred in 3 patients during a perioperative period. The patients were followed up for 6-24 months, and none suffered recurrence or metastasis.

**Conclusion:**

The efficacy of noninflating video-endoscopic ILND is good. Patients who have undergone the surgery not only have few postoperative complications but also have a good prognosis, suggesting the safety and availability of the clinical application.

## 1. Introduction

Penile carcinoma, with lymphatic metastasis as the main mode of metastasis, has an incidence as high as 10% in developing countries such as Asia and Africa [1]. Inguinal lymph node is not only the first position of metastasis and spread but also the related prognostic factors of penile carcinoma [2]. Open inguinal lymph node dissection (ILND) is a palliative treatment, which has good efficacy for nonmetastatic penile carcinoma [3, 4]. The National Comprehensive Cancer Network and European Urological Association guidelines have provided recommendations for the application of ILND in patients with penile carcinoma. However, the compliance is low [5, 6]. Besides, after ILND, the complication rates of the patients are 40-70% (such as infection, wound healing, prolonged lymphatic drainage time, lower limb edema, and scrotal lymphedema) [7]. The serious risks of the occurrence of ILND complications are still not resolved completely although various techniques have been improved and applied [8].

With the development of technologies, video-endoscopic ILND, which can maintain cancer treatment effect and reduce postoperative complications, has been applied gradually in clinic [9]. Studies have reported that patients treated with video-endoscopic ILND have lower complications than those treated with open ILND [10]. Tobias-Machado [11] performed bilateral ILND in a group of patients with high-risk inguinal lymph node metastases. Specifically, a standard open surgery was conducted on one side, and a video-endoscopic surgery was on another side. According to the results, it is found that, after the surgery, the incidence of complications in the open group was 70%, and that in the video-endoscopic group were 20%; and ski- related complications were 50% in the open group, 0 in the video endoscopic group. There was no significant difference in positive rate and total removed number of lymph nodes between the two groups. Additionally, video-endoscopic surgery had some advantages including magnified field of view, more refined operation, extensive use of energy instruments like ultrasonic scalpel, slight operation wound, and quick recovery. Compared with traditional open surgery, video endoscopic surgery can reduce surgical complications, which has obvious advantages. Unlike conventional laparoscopic surgery, video-endoscopic ILND requires the artificial construction of available subcutaneous space to contribute to the formation of subcutaneous and scrotal emphysema after the filling of CO_2_. Then under some pressure, the human body is promoted to absorb the CO_2_. However, the absorption of the CO_2_ causes a series of physiological state changes in the body, thereby affecting the blood gases, acid-base balance, respiratory system, hemodynamics, kidney physiology, and immune system, and even may promote the spread of tumor cells [12]. Some studies have shown that maintaining a low CO_2_ pressure during surgery does not increase the difficulty of surgery and can reduce hypercapnia caused by CO_2_ absorption [13].

Therefore, in order to avoid complications caused by CO_2_, this study innovatively utilized a noninflating suspension technique to conduct video-endoscopic ILND, providing an effective research direction and data support for the treatment and prognosis of penile carcinoma clinically.

## 2. Materials and Methods

### 2.1. Patient Collection

Eight patients with penile carcinoma who underwent noninflating video-endoscopic ILND in our hospital from May 2019 to March 2021 were collected, including 6 cases who were pathologically diagnosed by preoperative biopsy and underwent simultaneous partial penectomy and video-endoscopic ILND and 2 cases who underwent partial penectomy in the first stage and video-endoscopic ILND in the second stage. All patients signed informed consent, and this study was approved by Ethics Committee of the First Affiliated Hospital of Anhui Medical University.

### 2.2. Treatment Method

**Figure 1:**
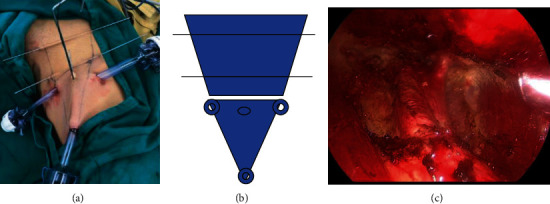
Diagram of patient undergoing non-inflating video-endoscopic inguinal lymph node dissection. (a) Layout of suspension and distribution diagram of puncture cannula; (b) surgical diagram; (c) surgical operating space.

In the supine position, the lower limbs of patients were separated by about 30 degrees to maximize exposure of the surgical space. The line between the upper edge of the external ring and the anterior superior iliac spine was considered upper boundary; the line between the anterior superior iliac spine and its lower 20 cm was the external boundary; the medial side of the pubic tubercle and its lower 15 cm was the internal boundary; the line between the internal boundary and the lower edge of the external boundary was the area that the lower boundary marked to be dissected. Then, subcutaneous working space was established and the puncture cannulas were indwelt. Specifically, Kirschner wires were placed subcutaneously at upper 1/3 and lower 1/3 of the operative region to be acted as two suspended planes. Then, the operating space for subcutaneous surgery without inflation was established through the application of self-made suspension retractor of Kirschner wire between puncture cannulas (Figures 1(a) and 1(b)). After being dissected from the region of the apical horn of the femoral triangle, the great saphenous vein and surrounding lymphoadipose tissues were lifted and freed toward the head. Further, the superficial inguinal lymph nodes within the above marked area were dissected. Specifically, the lymph nodes around the inguinal ligament were dissected upward until below the notch ligament and inguinal ligament. After superficial lymph node dissection, the Lata fascia was incised from the foramen ovale to expose the vascular and neural structures in the femoral canal, and then the femoral artery, femoral vein, and femoral nerve were observed (Figure 1(c)). Subsequently, lymphoadipose tissues around the femoral vessels were removed, and deep inguinal lymph nodes were dissected. After surgery, methylene blue solution was injected to observe and identify the lymphatic stump, and a drainage tube was indwelt for vacuum aspiration.

## 3. Results

### 3.1. General Information of Patients

Eight patients were aged 44-67 years, including 3 cases at clinical staging of T1cN0M0, 2 cases at T1cN1M0, 1 case at T2cN1M0, 1 case at T3cN1M0, and 1 cases at T3cN2M0 (Table 1).

### 3.2. Good Efficacy of Noninflating Video-Endoscopic Inguinal Lymph Node Dissection

All patients (*n* = 8) finished bilateral noninflating video-endoscopic ILND successfully. The surgical duration was 105-145 min, the hemorrhagic volume was 25-50 mL, the indwelling time of drainage tube was 5-12 days, and the hospital stay was 9-21 days. Left side lymph nodes were dissected for 3-14, and all were negative; and right side lymph nodes were dissected for 2-17, including one positive. All patients had no severe intraoperative complications such as massive bleeding. However, there were 3 cases of complications during the perioperative period, including 1 case of unilateral lymphorrhea, 1 case of bilateral lymphorrhea, and 1 case of unilateral lower limb edema. After a follow-up of 6-24 months after surgery, none of the patients suffer tumor recurrence or metastasis (Table 2).

## 4. Discussion

Penile carcinoma metastasis is performed through the lymphatic route. Specifically, the metastasis first occurs in inguinal lymph nodes and then spreads to the iliac paravascular lymph node. Inguinal lymph node metastasis and the degree of metastasis are one of the most important prognostic factors for penile carcinoma [14]. Some studies [15] have reported that the 5-year survival rate is about 80% for patients with 1-2 lymph node metastases; about 30% for patients with more than two metastases; and about 15% for patients with extralymphatic invasion, or the diameter of metastatic lymph nodes > 4 cm, or pelvic lymph node metastasis. Additionally, 25% of patients with inaccessible lymph nodes actually have occurred micrometastases, and once micrometastases develop into clinical lesions, the surgical cure rate will be significantly declined. ILND is not only an important treatment for patients with penile carcinoma but also the gold standard for the treatment of inguinal lymph node metastasis. However, the incidence of postoperative complications after traditional open ILND is as high as 50%-70%. And traditional open ILND is prone to cause flap necrosis, lymphatic fistula, lymphocele, delayed union, or even nonunion, which seriously affects the quality of life of patients [16]. Recently, the reports on video-endoscopic ILND have gradually increased [17]. Nevertheless, CO_2_ gas assistance is required during video-endoscopic ILND. And prolonged CO_2_ application during surgery in the abdominal cavity can cause subcutaneous emphysema and hypercapnia, which seriously affect the prognosis of patients [18].

In this study, noninflating technology was applied innovatively, and through the use of suspension method, video-endoscopic ILND was completed successfully without the filling of CO_2_. This operation does not require special equipment. The head frame that is always available in the operating room is used as a suspension bracket, and the Kirschner wire is percutaneously punctured to form a “two lines and one point” suspension plane, which is easy to operate. Further improvement of the suspension layout may increase the surgical operation space, which is an issue that we need to study further in the future. Use a bandage or silk thread to pull the suspension, and adjust the tightness of the bandage during the operation to obtain a satisfactory operating space. During the operation, the ultrasonic scalpel will generate a lot of smoke to interfere with the field of vision, and continuous suction is required to maintain a clear field of vision. We directly connect the negative pressure suction tube to the Trocar in the operation hole and use the observation hole and another operation hole to take in air. The sealing ring of the Trocar is removed to make the air circulate smoothly; the smoke generated by the operation can be quickly discharged. At the same time, the influence of the exhaust gas and smoke on the health of medical staff is avoided. There is no risk of subcutaneous emphysema, hypercapnia, and carbon dioxide embolism that may be caused by CO_2_ inflation. Eliminate concerns that inflation may lead to tumor dissemination and the impact on the body. All cases (*n* = 8) of noninflating video-endoscopic ILND (16 sides in total) we finished were successful, and the space established by suspension method could meet the surgical requirement. The number of lymph nodes dissected is similar to that dissected by conventional air-assisted laparoscopic surgery [19]. Additionally, the complications of patients during the perioperative period were recovered in a short time, and no serious complications occurred (such as cutaneous necrosis and deep venous thrombosis). The incidence of complications is not higher than other surgical methods reported in the literature [20, 21]. During postoperative follow-up, no recurrence or metastasis occurred, indicating good effect and high safety of the operation.

## 5. Conclusion

Noninflating video-endoscopic ILND has adequate surgical space, definite efficacy, few complications, and low recurrence rate for the treatment of penile carcinoma and can avoid side effects caused by the filling of CO_2_ in traditional video-endoscopic surgery. However, the number of patients included in this study is not sufficient, and the sample size needs to be increased for further study.

## Figures and Tables

**Table 1:** General information of patients.

Patients	Age (year)	Staging
1	56	T1cN1M0
2	65	T2cN1M0
3	63	T1cN0M0
4	53	T3cN2M0
5	62	T1cN0M0
6	44	T1cN0M0
7	67	T1cN1M0
8	53	T3cN1M0

**Table 2:** Evaluation of postoperative clinical indicators of patients.

Patients	Surgical duration (min)	Hemorrhagic volume (mL)	Drainage tube time (day)	Hospital stay (day)	Right side lymph nodes (positive/total)	Left side lymph nodes (positive/total)	Complication
1	125	50	5	11	0/6	0/8	No
2	138	45	12	21	0/12	0/10	Unilateral lymphorrhea
3	110	40	6	14	0/4	0/14	No
4	145	50	6	9	1/17	0/12	No
5	115	30	5	16	0/6	0/3	No
6	105	40	5	15	0/2	0/7	No
7	130	50	6	12	0/2	0/6	Unilateral lower limb edema
8	135	25	11	21	0/9	0/11	Bilateral lymphorrhea

## Data Availability

The data used to support the findings of this study are available from the corresponding author upon request.
